# Phylogenetic analyses suggest reverse splicing spread of group I introns in fungal ribosomal DNA

**DOI:** 10.1186/1471-2148-5-68

**Published:** 2005-11-21

**Authors:** Debashish Bhattacharya, Valérie Reeb, Dawn M Simon, François Lutzoni

**Affiliations:** 1 Department of Biological Sciences and Roy J. Carver Center for Comparative Genomics, University of Iowa, 446 Biology Building, Iowa City, IA 52242-1324, USA; 2 Department of Biology, Duke University, Durham, NC 27708-0338, USA; 3 Department of Biological Sciences, University of Calgary, Calgary, Alberta T2N 1N4, Canada

## Abstract

**Background:**

Group I introns have spread into over 90 different sites in nuclear ribosomal DNA (rDNA) with greater than 1700 introns reported in these genes. These ribozymes generally spread through endonuclease-mediated intron homing. Another putative pathway is reverse splicing whereby a free group I intron inserts into a homologous or heterologous RNA through complementary base-pairing between the intron and exon RNA. Reverse-transcription of the RNA followed by general recombination results in intron spread. Here we used phylogenetics to test for reverse splicing spread in a taxonomically broadly sampled data set of fungal group I introns including 9 putatively ancient group I introns in the rDNA of the yeast-like symbiont *Symbiotaphrina buchneri*.

**Results:**

Our analyses reveal a complex evolutionary history of the fungal introns with many cases of vertical inheritance (putatively for the 9 introns in *S. buchneri*) and intron lateral transfer. There are several examples in which introns, many of which are still present in *S. buchneri*, may have spread through reverse splicing into heterologous rDNA sites. If the *S. buchneri *introns are ancient as we postulate, then group I intron loss was widespread in fungal rDNA evolution.

**Conclusion:**

On the basis of these results, we suggest that the extensive distribution of fungal group I introns is at least partially explained by the reverse splicing movement of existing introns into ectopic rDNA sites.

## Background

Group I introns are autocatalytic RNAs that are widespread in organellar and nuclear genomes of eukaryotes, in eubacteria, and in phages and viruses [reviewed in [1-4]]. How these elements "move" within and between genes and between natural populations and species is poorly understood [4,5]. Two mechanisms are invoked to explain group I intron spread. The first is homing and is initiated by an intron-encoded endonuclease (homing endonuclease gene [HEG]) that recognizes and cleaves an intron-less allele at or near the intron insertion site [reviewed in [6]]. Following endonuclease cleavage at a specific 15 – 20 nt target sequence, the intron-containing allele is used as the template in a double-strand break repair pathway resulting in insertion of the intron and co-conversion of flanking exon sequences [7,8]. HEGs appear to be recurrently gained, degenerate, and lost in a cyclical manner and the intron-HEG combination is eventually lost from a population after all individuals are fixed for these elements [9]. A recent analysis in our lab of HEGs in nuclear rDNA group I introns showed that these coding regions are mobile elements (as has been shown for many organellar group I introns [reviewed in [6]]) that move either to introns in homologous rDNA sites or to introns in neighboring sites in often evolutionarily distantly related species [4,10,11]. The invading HEGs can then mobilize their new intron partners and achieve rapid spread within populations. However, our in-depth phylogenetic analyses of the HEGs failed to show the involvement of these endonucleases in the movement of group I introns into distant rDNA sites [10]. This type of long-distance (e.g., >50 nt) movement has, however, been suggested by phylogenetic studies that show group I introns from sites such as SSU rDNA S287 and S1199 (numbering based on the *Escherichia coli *gene) to be closely related [12].

**Figure 1:**
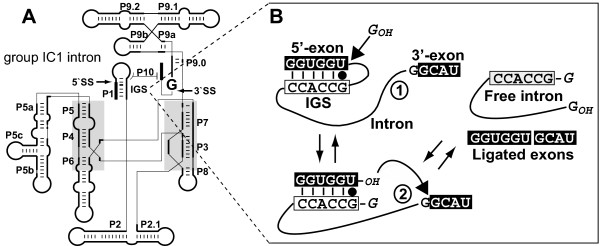
**Group I intron splicing**. A) The typical secondary structure of a group I intron which consists of about ten paired elements (P1–P10). The intron internal guide sequence (IGS) is shown that recognizes the 5' exon sequence through a 4–6 nt base pairing, thereby initiating the two-step splicing mechanism (shown in panel B) for group I intron removal from pre-RNA. B) The forward and reverse splicing of a group I intron is shown in this figure. The arrows that lead from the free intron to the pre-RNA indicate the reverse splicing reaction. Both forward and reverse splicing reactions require the IGS interaction in domain P1.

Long-distance intron movement can in principle be achieved by reverse splicing that facilitates intron mobility through an RNA intermediate [13-16]. In reverse splicing, group I introns recognize their target sequence through complementary base pairing with a short (4–6 nt) internal guide sequence (see Fig. 1) followed by integration into the transcript [e.g., [14]] and then putatively reverse-transcription, and general recombination to achieve spread. The importance of this pathway in group I intron movement in nature, however, remains to be established because reverse splicing-mediated intron movement has not been demonstrated in genetic crosses. Furthermore, whereas homing is highly efficient in spreading introns in populations, it is likely that reverse splicing with its reliance on chance integration followed by two additional steps (i.e., reverse-transcription, recombination) would be less efficient in promoting intron movement. An additional constraint is that rDNA exists as a multi-copy gene family necessitating that alleles containing transferred introns must rise to high frequency (presumably through concerted evolution or less parsimoniously, repeated reverse splicing events) in individuals and in populations to ensure survival and ultimately, fixation. If group I introns are weakly deleterious, then fixation may occur only in species with small population sizes [17]. These considerations suggest that rare reverse splicing events may be most successfully recognized in the context of broadly sampled host and intron phylogenies in which many potential candidates for reverse splicing movement are studied. The large collection of fungal group I introns that has recently accumulated provides an ideal opportunity to test comprehensively the contribution of reverse splicing to the extant intron distribution.

To assess whether phylogenetic evidence exists that is consistent with a reverse splicing mechanism of group I intron spread, we analyzed a large group I intron data set (189 sequences) from the Pezizomycotina. These fungi are particularly intron-rich with some taxa containing up to 10 ribozymes in the rDNA (e.g., *Physconia perisidiosa*). In addition, we included 10 rDNA group I introns from the yeast-like fungal symbiont of anobiid beetles, *Symbiotaphrina *spp. [18]. We addressed two questions with this study: 1) Is there phylogenetic evidence for the reverse splicing-mediated spread of group I introns in the Pezizomycotina fungi? 2) Are the multiple group I introns in *Symbiotaphrina *spp. that are shared with other fungi the result of independent lateral transfers into the rDNA genes of these taxa, or have some or all of these introns been vertically inherited in the fungi? For the latter question we were particularly interested in determining whether *Symbiotaphrina*, which lives in the gut of beetles and could therefore theoretically come in contact with many different cells, be a potential vector for group I intron spread. Although many studies have provided evidence for the movement of group I and group II introns across broad evolutionary lines [e.g., [5,12,19-22]], there is little known about the vectors that facilitate their spread. Our analyses demonstrate a complex evolutionary history of the fungal introns with many cases of vertical inheritance (putatively for the 9 introns in *S. buchneri*) and intron lateral transfer. In addition, there are several examples in which introns, many of which are still present in *S. buchneri*, may have spread through reverse splicing into heterologous rDNA sites.

## Results and discussion

### General patterns of fungal group I intron inheritance

**Figure 2:**
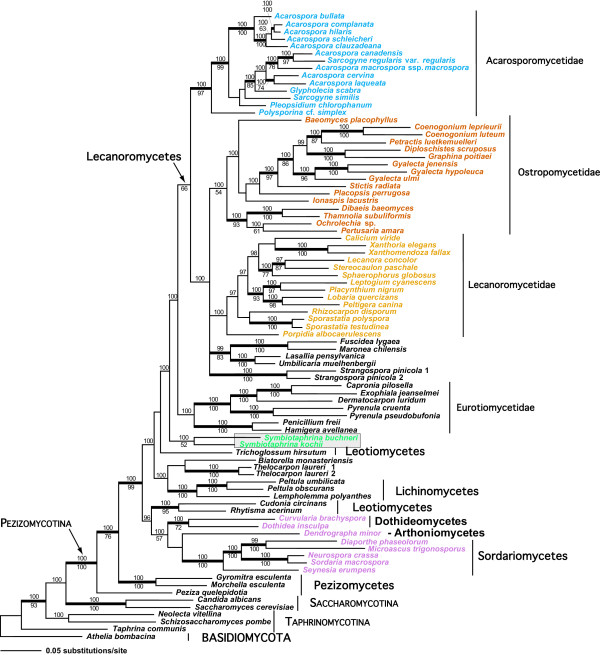
**Host tree of fungi**. Bayesian and NJ analyses of fungi showing the position of the genus *Symbiotaphrina *within the Ascomycota. This tree is inferred from a combined data set of nuclear SSU rDNA, LSU rDNA, and *RPB*2 from 84 species of the Ascomycota with one basidiomycete species used as the outgroup. The phylogram represents the majority rule consensus tree of 40,000 post-burnin trees sampled by the Bayesian search algorithm. The lengths for each branch were averaged over all trees having this branch (sumt option in MrBayes v30b4). Numbers above internodes are posterior probabilities (when ≥95%). Values below the internodes are NJ bootstrap proportions. If both the Bayesian posterior probabilities are ≥95% and the NJ bootstrap support are ≥70%, the internal branch is shown as a thicker line. The grey box delimits the genus *Symbiotaphrina *(bluish green text). Supra-generic taxon names follow [49]. The major intron-containing fungal groups are shown in different colors (Acarosporomycetidae in blue, Ostropomycetidae in vermillion, Lecanoromycetidae in orange, and the Sordariomycetes in reddish purple).

We took advantage of the most group I intron-rich of all eukaryotes, the Pezizomycotina fungi (in particular, the lichen-forming fungi [12,23]), to address the movement and long-term evolution of these mobile elements. The nuclear group I introns are found exclusively in rDNA genes with some taxa containing 7 (*Gymnoderma coccocarpum*), 8 (*Diplotomma epipolium*), 9 (*Symbiotaphrina buchneri*), or 10 insertions (*Physconia perisidiosa *[12,23]) in their SSU and LSU rDNA. To facilitate the analysis of fungal group I introns, we first inferred a "host" tree of the Pezizomycotina based on the analysis of SSU and LSU rDNA + *RPB2 *data (Fig. 2). The rDNA sequences for the four *S. kochii *strains were identical (each encoded a single group I intron at position L1921). The rDNA coding regions of *S. buchneri *JCM9740 were interrupted by 5 introns in the SSU rDNA coding region (S114, S287, S1052, S1210, S1506) and by 4 introns in the LSU rDNA (L1094, L1921, L2066, L2449). In the host tree, *S. buchneri *and *S. kochii *form a clade with Bayesian but not bootstrap support, providing weak evidence for their monophyly (see grey box in Fig. 2). This result was previously found in a more limited analysis of partial small subunit rDNA sequences [24]. In our tree, the *Symbiotaphrina *species diverge before the split of most of the major lineages of lichen fungi (i.e., Lecanoromycetes + Eurotiomycetes), but again without bootstrap support. If the early divergence of *Symbiotaphrina *is correct, then the fungal host tree suggests that under a model of vertical inheritance, in intron trees, the nine *S. buchneri *group I introns should each form independent monophyletic groups containing other fungal introns at the respective insertion sites. The *S. buchneri *sequences should not cluster on the basis of their common occurrence in this species and ideally, they should branch after the Sordariomycetes but before the Lecanoromycetes + Eurotiomycetes group (see Fig. 2).

**Figure 3:**
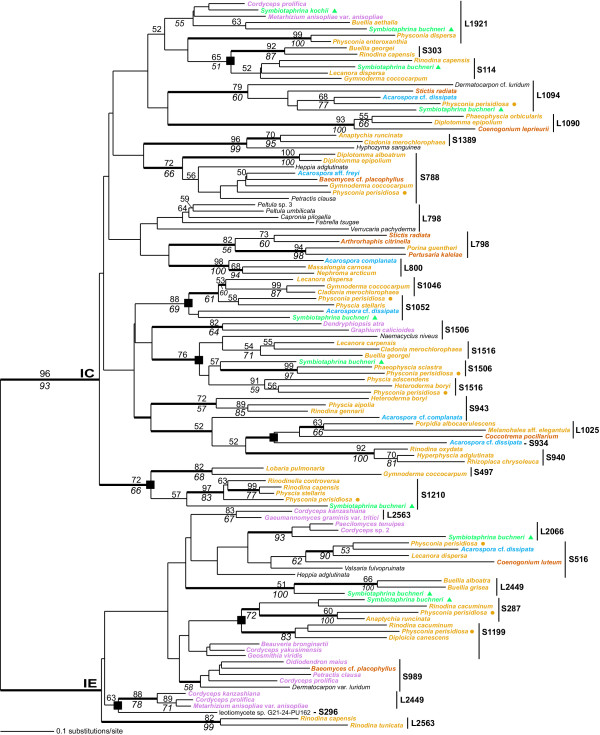
**Phylogeny of group I introns**. Phylogeny of fungal group I introns implicated in reverse splicing movement. This is a JC-NJ tree that was inferred for 116 fungal introns. The results of a JC-NJ bootstrap analysis are shown above the branches, whereas the results of an unweighted maximum parsimony bootstrap analysis are shown below the branches. The thick branches represent ≥95% Bayesian posterior probability. Branch lengths are proportional to the number of substitutions per site (see legend). The *Symbiotaphrina *spp. group I introns are marked with the filled triangles and the *P. perisidiosa *introns are marked with the filled circles. The intron insertion sites in small (S) and large (L) subunit rDNA are shown. The filled squares at the nodes denote the putative cases of intron movement. The colors for the different introns reflect the taxonomic position of the host cell containing the ribozymes (consistent with the scheme shown in Fig 2).

Given these expectations, we first analyzed the 189-sequence intron data set that was used as input for the JC-NJ and Bayesian inference of phylogeny (results not shown). This tree was consistent with a model of vertical inheritance with the fungal introns assorted primarily on the basis of their sites of rDNA insertion and not intermixed, which would result if they had been frequently laterally transferred to ectopic sites ([3,10,12,22,25] for exceptions, see below). A JC-NJ analysis using a subset of 116 sequences provided the same results (Fig. 3) but with higher bootstrap support due to the reduced number of taxa in the data set. In particular, the nine *S. buchneri *group I introns (green filled triangles in Fig. 3) are distributed in the tree on the basis of their site of rDNA genic insertion, often in clades with bootstrap and/or Bayesian support. The same holds for the *P. perisidiosa *group I introns (yellow filled circles) in Fig. 3. These results argue for a separate evolutionary history for the different introns although within each intron lineage there could be a combination of vertical inheritance and lateral transfer between fungi [e.g., [10]]. This would result if some of the introns were targeted (i.e., fixed) at the homologous rDNA site in other species resulting in their monophyly; i.e., supporting intron vertical inheritance even though the ribozymes had moved between species. This phenomenon is best addressed with targeted analyses of specific intron lineages and the fungi that contain these introns [e.g., [23,25]].

**Figure 4:**
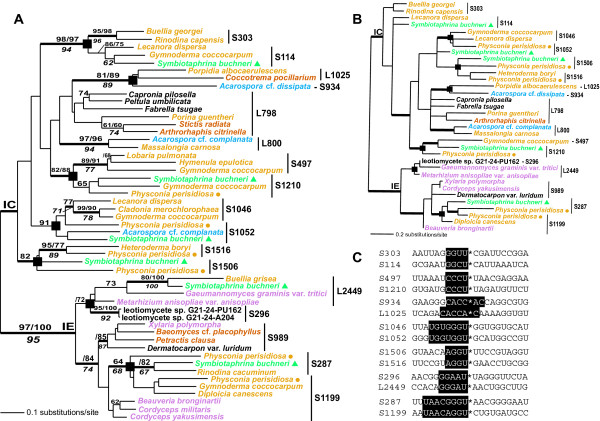
**Testing putative cases of group I intron reverse splicing**. Phylogeny of fungal introns and analysis of rDNA flanking exons to assess putative cases of group I intron reverse splicing. A) Distance matrix phylogenetic tree of a reduced data set of 51 fungal introns. The results of a distance bootstrap analysis are shown above the branches on the left of the slash marks, whereas the results of a maximum likelihood bootstrap analysis are shown on the right of the slash marks. The values shown below the branches result from an unweighted maximum parsimony bootstrap analysis. The thick branches represent ≥95% Bayesian posterior probability. Branch lengths are proportional to the number of substitutions per site (see legend). The *S. buchneri *group I introns are marked with filled triangles and the *P. perisidiosa *introns are marked with the filled circles. The putative cases of intron movement are denoted with the filled squares on the internal nodes. The rDNA intron insertion site is shown for each ribozyme. B) Majority-rule consensus tree inferred from a Bayesian analysis of 34 fungal group I introns. Only the core regions of the ribozymes were used in this analysis. The colors for the different introns in panels A and B reflect the taxonomic position of the host cell containing the ribozymes (consistent with the scheme shown in Fig 2). C) Alignment of exon sequences (all from *S. buchneri*) at heterologous group I intron sites. Regions required for the IGS interaction that are implicated in reverse splicing intron movement are shown in the boxed areas.

In general the introns in *S. buchneri *are either limited to the Lecanoromycetes (S114, S1052, S1210) or are more closely related to homologs in this group than in the Sordariomycetes, as would be expected under a model of vertical inheritance (e.g., L1921, L2449). The single L1921 group I intron in *S. kochii *appears however to have an origin through lateral transfer from a sordariomycetes source (albeit without bootstrap support, Fig. 3). We tested the hypothesis of independent origins of the *S. buchneri *group I introns by forcing the monophyly of these sequences from different sites in the tree inferred from the 51-sequence data set (see Fig. 4). The Shimodaira-Hasegawa statistical test significantly rejected trees, for example, in which the *S. buchneri *S1210 and S114 (S1210 branch moved to S114, or S114 branch moved to S1210, P < 0.01), the S287 and L2449 (S287 to L2449, or L2449 to S287, P < 0.001), or the S114 and S1506 (S114 to S1506, or S1506 to S114, P < 0.001) introns were united in one clade. Forcing the monophyly of any of the *S. buchneri *IC and IE introns resulted in the greatest differences in log likelihood arguing for a long evolutionary separation of these intron subgroups. The only tree rearrangement that did not result in significant SH-test scores was for the union of the *S. buchneri *S1052 and S1506 group I introns (S1052 to S1506, P = 0.153; S1506 to S1052, P = 0.195). This suggests a potential common evolutionary origin of these introns.

In summary, our data are consistent with the idea that the *S. buchneri *group I introns have independent origins and that they been vertically inherited in many fungi. Our data argue most strongly for the view that the *S. buchneri *introns have not recently spread in rDNA through ectopic transposition within this taxon. Consistent with these ideas, detailed analysis of the Sordariomycetes support the hypothesis of long-term group I intron vertical evolution [25]. We cannot, however, unambiguously ascertain the extent of intron lateral transfer between all Pezizomycotina because of the uneven and sporadic distribution of the data (i.e., the Sordariomycetes and Lecanoromycetes contain the majority of fungal group I introns. If the *S. buchneri *introns are ancient as we postulate, then group I intron loss was widespread in fungal rDNA evolution because these sequences are not present in many fungi (e.g., completely absent, to date, in the Dothideomycetidae and rare in the Eurotiomycetidae [Reeb et al. unpublished data]). Within the Lecanoromycetidae, detailed analysis of this group using SSU rDNA comparisons shows that entire derived lineages (e.g., Bacidiaceae, Peltigeraceae, Rhizocarpaceae) are intron-free even though their sisters often contain multiple different group I introns (e.g., Acarosporaceae [Reeb et al., unpublished data], Cladoniaceae, Physciaceae [12,23]).

### Evidence for group I intron spread

Although group I introns form monophyletic groups in Figure 3, there are also cases of a close relationship (bootstrap and/or Bayesian support) between intron clades at ectopic rDNA sites. Inspection of Figures 3 and 4 suggests seven potential transposition events (marked with filled boxes). These same cases were also present in the 189-sequence data set (results not shown). The intron movements involve four cases that were previously found in more limited phylogenetic analyses of clades S114 – S303, S1046 – S1052, S287 – S1199 (without bootstrap or Bayesian support in Fig. 3, but see Fig. 4A), S1506 – S1516 [12] and three putative new cases found in this study (S497 – S1210, S934 – L1025, S296 – L2449 [again, see Fig. 4A]). Perhaps most interesting in this analysis is the finding that five of the nine *S. buchneri *SSU rDNA introns (S114, S287, S1052, S1210, S1506) are nested inside, or sister to introns at heterologous sites in the Lecanoromycetidae (see Figs. 3 and 4). This suggests that the five potentially ancestral SSU rDNA introns that were present in *S. buchneri *may have spread into 5 novel sites (i.e., S303, S1199, S1046, S497, S1516, respectively) during fungal evolution resulting in 10 different intron lineages. The timing of these intron movements suggests that most have occurred in derived members of the Lecanoromyectidae, in particular the lichenized Physicaceae [12]. The S1052 to S1046 movement, for example, must have been relatively recent because, despite extensive sampling [12,23], the novel S1046 intron is known only from the closely related taxa, *Gymnoderma coccocarpum*, *Physcia stellaris*, *Lecanora dispersa*, and *Cladonia *spp. (Lecanorales). The S114 to S303 movement is also likely to be recent because the derived S303 intron is limited to two members of the Physciaceae (*Buellia georgei *and *Rinodina cacuminum*). Other intron movements that are independent of *S. buchneri *include the S934 – L1025 and S296 – L2449 (see Figs. 3 and 4A,4B) sites. These groupings are only weakly supported in Fig. 3. All of the phylogenetic analyses with the 51-sequence data set suggest however that the *Acarospora *cf. *dissipata *S934 group I intron shares a specific evolutionary relationship with the intron at the L1025 site in a member of the Lecanoromycetidae (*Porpidia albocaerulescens*) and the Ostropomycetidae (*Coccotrema pocillarium *[Fig. 4A]). The majority-rule Bayesian consensus tree inferred from the intron core regions is consistent with these results showing that the intron transpositions are supported even when a restricted number of sites from the most highly conserved regions of the ribozymes are used in the analysis (Fig. 4B). This result argues against the spurious clustering of introns in Figures 3 and 4A resulting solely from long-branch attraction of divergent sequences.

Taken together, our phylogenetic analyses provide evidence not only for the ancient origin of many fungal introns that can be traced back to their presence in *S. buchneri*, but also provide indirect evidence for the spread of some of these introns into novel sites, thereby giving rise to additional vertically inherited sequences. Given this hypothesis, we predict that some taxa may maintain introns at both the ancestral and novel sites. This prediction is met for the S114 – S303 (*Rinodina capensis*), S287 – S1199 (*Physconia perisidiosa*, *R. cacuminum*), and S1506 – S1516 introns (e.g., *P. perisidiosa*). The low number of taxa containing both ancestral and derived introns suggests again that intron loss is widespread in fungal rDNA because in most cases one or both of the ribozymes have been lost from the genes. We cannot yet identify, however, the source of many introns that are putatively of a putative recent origin but are unrelated to the introns in *Symbiotaphrina *(e.g., S1389, L800, L1090). Clearly, external sources such as viruses or bacteria may also play an important role in the introduction of group I introns in fungal rDNA.

### Reverse splicing group I intron spread

We suggest that reverse splicing may be an important mechanism of group I intron spread into distant rDNA sites in fungal nuclear rDNA for the following reasons: 1) Virtually none of the Pezizomycotina rDNA group I introns (including all of those implicated here in movement) contain endonucleases [10,26,27]. If endonucleases (encoded within the intron sequences) did mediate the lateral transfer of rDNA introns, then one would have to postulate complete loss of these coding regions after the introns had attained their present distribution. When endonucleases have been found in nuclear group I introns [see [10]], they appear to have been inserted into existing intron sequences and generally do not contain extensive deletions but rather frame-shift mutations or short truncations that result in their inactivation [e.g., [26,28], Reeb et al. unpublished data]. No large insertions (i.e., >200 nt) have been found in these introns that could be the remnant of an inactivated endonuclease open reading frame. 2) Generally, intron homing requires extensive exon flanking sequence identity (15 – 20 nt [2,7,8]) that is not found among the different rDNA group I intron movements (Fig. 4C). 3) In the cases of intron transposition shown here, all of the heterologous rDNA sites show high conservation of only 4 – 8 nt of the 5' exon flanking sequence (all exon sequences are from *S. buchneri *rDNA). This region is involved in the internal guide sequence interaction that is required for both forward- and reverse splicing (Fig. 1). In our analyses, the S287 – S1199 and S1046 – S1052 introns share the 5' flanking exon sequences ^5'^UAACRGGU^3' ^and ^5'^UGKKGGU^3'^, respectively (Fig. 4C). A close evolutionary relationship between the S497 – S1210 introns is also supported by this analysis (^5'^CCCU^3'^). This pattern of sequence conservation is predicted for reverse splicing-mediated intron spread [13,15]. A recent analysis of the S956 twin-ribozyme in the myxomycete *Didymium iridis *demonstrates that it can accurately reverse-splice into the homologous site in both *E. coli *and yeast rRNA [29]. Surprisingly, this intron does not integrate into related heterologous rRNA sites as has been reported for the *T. thermophila *ribozyme, which was shown to partially reverse-splice into 69 sites and completely integrate into one site in *E. coli *large subunit rRNA [16]. The S934 – L1025 intron group also displays a conserved motif at the site of insertion (^5'^CACCAC^3'^) but one of these introns appears to have "slid" along the exon by one nucleotide either at the time of reverse splicing or sometime thereafter.

## Conclusion

Given the results of our exhaustive study, we propose that reverse splicing is potentially an important mechanism of intron spread in Pezizomycotina nuclear rDNA. Our analyses provide strong support for the idea that the evolutionary history of nuclear group I introns may differentiate itself markedly from organellar group I introns which appear to rely primarily on homing for spread and may be characterized by massive and independent invasions into the homologous DNA sites of related taxa [e.g., [10,30]]. In contrast, the nuclear introns often have protracted histories in the host genomes [e.g., [12,23,25,31]] and their spread into novel sites may be a direct consequence of this long-term stability; i.e., existing introns reverse-splice at different times into heterologous sites. The availability of reverse transcriptase in the cell and the fixation rate of intron-containing alleles in the multi-copy rDNA gene family are likely the primary determinants of the rate of reverse splicing-mediated movement. Limited analyses of intron^+ ^and intron^- ^strains fail to show any clear advantage or disadvantage to the host cell when introns are present [32,33]. This suggests that mobile group I introns may be "silent" parasites that have no measurable phenotype [34,35]. The neutral nature of group I introns suggests that their spread and loss may be stochastic events with rare movement occurring through reverse splicing and the more frequent intron loss occurring most likely through chance or when intron mobility, splicing, and/or processing (e.g., degradation) poses a cost to the host cell [7]. Successful group I intron excision is of critical importance because these sequences are normally found in functionally important regions of rDNA [36,37].

Our study suggests that the beetle gut symbiont *S. buchneri *[see [38]] contains a set of introns that has likely been vertically inherited in later-diverging fungi with reverse-splicing spread of some of these ribozymes into ectopic rDNA sites, particularly in the intron-rich Lecanoromycetes. Our data do not support the idea that *S. buchneri *is a vector for facilitating intron spread in the fungi. We did not find an unexpectedly close phylogenetic relationship between any of the introns in this taxon with others in our data set, which would have indicated a recent later transfer event. The phylogenetic positions of the *S. buchneri *introns relative to other ribozymes (when bootstrap and/or Bayesian support is found) are generally consistent with the expectations of host relationships. However, these group I intron data do not have enough resolving power to allow us to address the possibility of more ancient transfer events involving *S. buchneri*. In conclusion, it is apparent from our work that nuclear group I introns in the fungi have remarkably complex evolutionary histories, therefore our analyses are likely only scratching the surface of intron movement in these taxa. More detailed studies of specific intron lineages [e.g., [11,23,25]] will more accurately reveal the dynamics of fungal rDNA group I intron evolution.

## Methods

### Fungal cultures, DNA extraction, and PCR amplification

Five cultures of *Symbiotaphrina *spp. were acquired for this study: *S. buchneri *(JCM9740) and *S. kochii *(JCM9739, CBS250.77, CBS588.63, CBS589.63). DNA was isolated from these cultures using the Puregene Kit (Gentra Systems) following the manufacturer's protocol for filamentous fungi. The nuclear small (SSU) and large subunit (LSU) rDNA were amplified for each strain by PCR. In addition we amplified the *RPB*2 (second-largest subunit of RNA polymerase II) nuclear gene from *S. buchneri *JCM9740 and *S. kochii *CBS589.63. PCR products were purified from agarose gels using GELase (Epicenter) and directly sequenced on both strands using an ABI PRISM 3700 DNA Analyzer (Applied Biosystems), and Big Dye (Perkin-Elmer, Applied Biosystems). The PCR primers used in this study came from various sources [39-47] or were designed specifically for *Symbiotaphrina *spp. The rDNA and *RPB*2 sequences for *Symbiotaphrina *spp. are available from GenBank under the following accession numbers respectively: *S. buchneri *JCM9740 (rDNA = DQ248313; *RPB2 *= DQ248315), *S. kochii *CBS589.63 (rDNA = DQ248314; *RPB2 *= DQ248316).

### Sequence alignments and phylogenetic analyses

#### Fungal host phylogeny

To provide a framework for understanding group I intron evolution in the fungi, we reconstructed a phylogeny of the Pezizomycotina that included *Symbiotaphrina *spp. This tree was inferred from a combined DNA data set of nuclear SSU rDNA, LSU rDNA, and *RPB*2 DNA (2100 nt) from 84 ascomycetes with one basidiomycete as the outgroup. These data are available from GenBank. The combined data set was analyzed using a GTR + Γ + I model of evolution for each of the five data partitions (SSU, LSU, RPB2-1^st^, -2^nd^, -3^rd ^codon positions). Bayesian analysis (MrBayes V3.0b4 [48]) was initiated using a random tree from the combined dataset with four chains running simultaneously for 5,000,000 generations, and trees sampled every 100 generations. The first 10,000 trees were discarded (burnin) and a majority rule consensus tree was generated from the remaining 40,000 (post burnin) trees. A neighbor-joining analysis was also used to calculate bootstrap support values for nodes in the Bayesian consensus tree. The supra-generic taxon names used in this tree follow [49].

#### rDNA group I intron phylogeny

The ten group I introns found in *Symbiotaphrina *spp. were aligned with 179 fungal group I introns at 28 different rDNA sites. The non-*Symbiotaphrina *introns are published [e.g., [12]] and available either from GenBank or from the Comparative RNA Web Site [50]. The introns from the 28 rDNA sites represent well the diversity of fungal rDNA group I introns although we excluded a small number of introns from other sites that were either difficult to align or to unambiguously identify their rDNA genic position (e.g., S940, S1049, S1201). The 189 group I introns were aligned through juxtaposition of the secondary structural elements P1–P9 found in nuclear group I introns [31,51,52]. For this procedure we used, wherever possible, existing secondary structures from representatives of different intron insertion sites [e.g., [10,11,53-55]] to guide the alignment. We did not attempt to include all available fungal group I introns (there are nearly 1200 group I introns in this group [see [50]]) but sampled (given the taxonomic distribution) evenly the different lineages. Our approach was designed to provide an overall view of fungal group I intron phylogeny and is not expected to detect lateral transfers within intron lineages that would be apparent in detailed analyses of introns at particular rDNA sites and the host phylogeny [e.g., [10,11,20,23,25,55]]. Given the large number of introns and rDNA genic sites to consider, we divided the phylogenetic analyses into increasingly more focused data sets. The initial data set of 189 introns was used to gain broad insights into group I intron phylogeny and in particular, the distribution of the *S. buchneri *introns within the tree. This tree provided evidence for the vertical evolution and movement of introns. Thereafter, we reduced the data set to a representative group of 116 introns to increase the phylogenetic resolution. Finally, we included the putative reverse splicing candidates in data sets of 51 and 34 sequences. The sequences were pruned approximately uniformly from the trees to retain the diversity of introns at the different rDNA sites. This approach was necessary to gain meaningful insights into group I intron evolution because phylogenetic methods often perform poorly under the situations used here; i.e., the interrelationships of many divergent lineages need to be resolved with a relatively small data set.

A total of 136 aligned positions were selected for the initial phylogenetic analyses (alignment available from DB upon request). For these data, we used two different approaches to infer the phylogeny. First, we used the single parameter Jukes-Cantor (JC) evolutionary model [56] with neighbor-joining (NJ) tree reconstruction to estimate a tree. This "simple" model is potentially useful for large data sets with short (in this case, highly divergent) sequences when multiple parameter estimates are expected to have high associated variances [e.g., [57,58]]. Under such conditions, the maximum likelihood method may give an incorrect topology [59]. Branch lengths will, however, be underestimated under the JC model. The JC-NJ tree was inferred using PAUP* (V4.0b10 [60]) and bootstrap analyses (2000 replications) were done to assess the support for monophyletic groups in the JC-NJ tree. In the second approach, we used the parameter-rich GTR + Γ model ([61] i.e., estimated proportion of invariant sites = 0.0147) in a Bayesian inference as described above to calculate posterior probabilities for nodes in the intron tree. In this analysis, a random starting tree was initiated and run for 3,000,000 generations with trees sampled every 1000^th ^generation. To increase the probability of chain convergence, the first 2,000 trees were discarded as burnin and the remaining 1,000 were used to calculate the posterior probabilities.

Based on the analysis of the 189-sequence data set, we generated a second reduced intron data set of 116 sequences that maintained the diversity of intron sites in the large data set. A JC-NJ tree was inferred from these data (with bootstrap support values) and Bayesian posterior probabilities were calculated for the tree as described above. In addition, we did an unweighted maximum parsimony (MP) bootstrap analysis of the data. For this method, a heuristic search was used with each of the 2000 bootstrap pseudosamples and starting trees were obtained using random additions (10 rounds) with tree bisection-reconnection branch swapping. The 51-sequence data set (138 nt in this alignment) was analyzed with the JC-NJ, MP, and Bayesian methods as described above. In addition, we did a maximum likelihood bootstrap analysis of these data. In this approach, the gamma value (with 4 rate categories) and the transition/transversion ratio were estimated using PAUP*. Bootstrap analyses (100 replicates) were then done using DNAML (PHYLIP V3.6b [62]) with 1 random taxon addition and global rearrangements. We also generated a second reduced alignment of 34 introns that included only the catalytic core (66 nt) of the fungal group I introns [11]. Analysis of the core region alignment allowed us to assess whether the most highly conserved region of these ribozymes resulted in essentially the same tree as when the more variable regions were included. For the core alignment, we used Bayesian inference (as described above) to infer a 50% majority-rule consensus phylogeny from the final 1000 trees in the posterior distribution. In all of these intron phylogenies, the evolutionarily distantly related group IE introns [5,37] were used to root the subtree of IC introns [10]. Finally, we used the maximum likelihood-based Shimodaira-Hasegawa statistical test [63] to assess likelihood support alternative intron topologies.

## Authors' contributions

VR and FL did the sequencing of group I introns and did the phylogenetic analysis of the fungal host tree and contributed to the manuscript. DMS sequenced fungal introns and contributed to the manuscript. DB conceived of and supervised this study and wrote the manuscript. All authors read and approved the final manuscript.
